# 3D Electron Diffraction Structure Determination of Terrylene, a Promising Candidate for Intermolecular Singlet Fission

**DOI:** 10.1002/cphc.202100320

**Published:** 2021-06-30

**Authors:** Charlie L. Hall, Iryna Andrusenko, Jason Potticary, Siyu Gao, Xingyu Liu, Werner Schmidt, Noa Marom, Enrico Mugnaioli, Mauro Gemmi, Simon R. Hall

**Affiliations:** ^1^ School of Chemistry University of Bristol Bristol BS8 1TS UK; ^2^ Istituto Italiano di Tecnologia Center for Nanotechnology Innovation@NEST Pisa 56127 Italy; ^3^ Department of Materials Science and Engineering Carnegie Mellon University Pittsburgh Pennsylvania 15213 USA; ^4^ PAH Research 86859 Igling-Holzhausen Germany

**Keywords:** terrylene, electron diffraction, singlet fission, crystal, oligorylenes

## Abstract

Herein we demonstrate the prowess of the 3D electron diffraction approach by unveiling the structure of terrylene, the third member in the series of *peri*‐condensed naphthalene analogues, which has eluded structure determination for 65 years. The structure was determined by direct methods using electron diffraction data and corroborated by dispersion‐inclusive density functional theory optimizations. Terrylene crystalizes in the monoclinic space group *P*2_1_/*a*, arranging in a sandwich‐herringbone packing motif, similar to analogous compounds. Having solved the crystal structure, we use many‐body perturbation theory to evaluate the excited‐state properties of terrylene in the solid‐state. We find that terrylene is a promising candidate for intermolecular singlet fission, comparable to tetracene and rubrene.

## Graphical abstract

**Terylene for singlet fission**? 3D electron diffraction (ED) is used to overcome a gap in the crystallographic knowledge, by reporting the first structural solution of terylene. Cutting‐edge computational analysis show that terylene has the potential to act as an effective singlet fission chromophore. This represents a substantial milestone in evolution of 3D ED, by demonstrating that hitherto unobtainable structures can be used in the prediction of physical properties of functional materials.

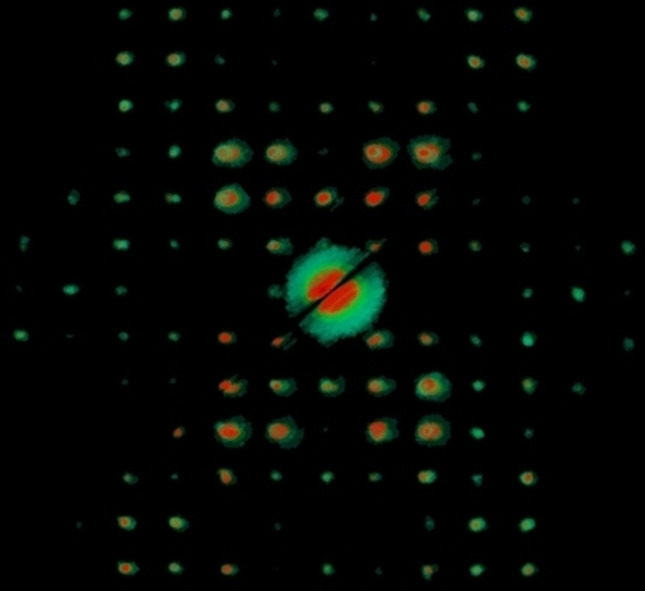

## 1 Introduction

The strong interaction of electrons with matter has attracted a growing interest since late 1900s, when it became evident that electron diffraction (ED) data acquired by a transmission electron microscope (TEM) can be used for the structure determination of nanocrystals.[^1^, ^2^, ^3^, ^4^] A crucial methodological breakthrough was the development of routines for sequential acquisition of 3D diffraction data, after tilting the sample in fixed angular steps around an arbitrary axis.[^5^, ^6^, ^7^, ^8^] The resulting data set can thus be used for the determination of cell parameters and Laue class, as well as for the integration of reliable reflection intensities.

Powder X‐ray diffraction (XRD) is a user‐friendly and easily accessible option for dealing with nano‐scaled crystals, but this method is substantially limited by the projection of the information onto only one dimension, which necessarily introduces ambiguities during data analysis of an unknown structure. Conversely, a 3D electron diffraction (3D ED) approach is conceptually comparable to single‐crystal XRD, but tolerates collecting data from much smaller volumes, in a range of about 10^0^–10^−4^ μm^3^.^[9]^ In recent years, 3D ED has been extensively used for the structure determination of various nanocrystalline materials, ranging from inorganics to macromolecules.[^10^, ^11^, ^12^, ^13^, ^14^, ^15^, ^16^, ^17^, ^18^] In the world of small‐molecule organic compounds, 3D ED has been successfully employed for unveiling the structure of organic frameworks,^[19]^ semiconductors^[20]^ peptides,[^21^, ^22^] pharmaceuticals[^23^, ^24^, ^25^, ^26^] and natural products,[^27^, ^28^, ^29^] all of which could not be addressed by XRD methods.

**Figure 1:**
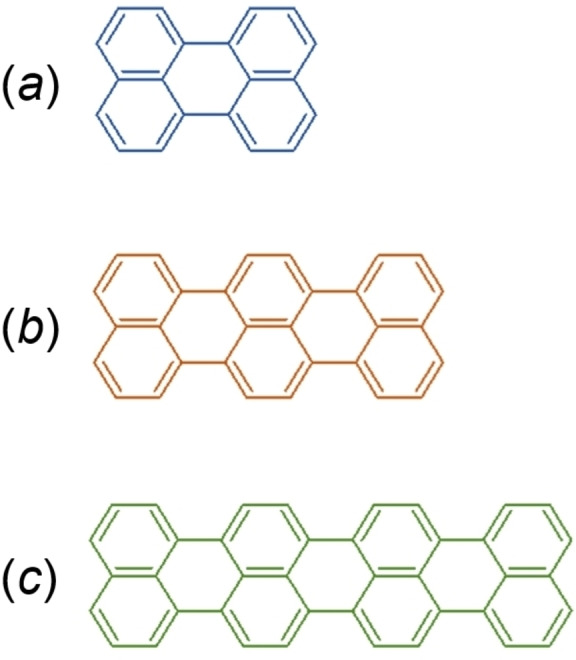
Molecules in the oligorylenes family discossed in this work: (a) perylene, (b) terrylene, (c) quaterrylene.

Terrylene (C_30_H_16_) is a rigid polycyclic aromatic hydrocarbon (PAH) consisting of eight fused aromatic rings.^[30]^ The sequence of aromatic rings follows that of the five rings of perylene (C_20_H_12_)^[31]^ and the eleven rings of quaterrylene (C_40_H_20_).^[30]^ These “condensed” ring systems (Figure 1), with unique thermodynamic and electronic properties, belong to the oligorylenes family,^[32]^ known also as poly(*peri*‐naphthalene)s.^[33]^

The second member of the oligorylenes family, perylene, has two known polymorphs. The crystal structure of α‐perylene was determined by Donaldson et al.,^[34]^ while β‐perylene was obtained and resolved by Tanaka.^[35]^ The crystal structure of quaterrylene was first inferred on the basis of two dimensional XRD data^[33]^ and later re‐determined with three dimensional XRD data).^[36]^ In quaterrylene and α‐perylene, the crystal structures consist of parallel pairs of nearly planar molecules, related one to another by a centre of symmetry (dimers) and arranged in a sandwich‐herringbone (SHB) motif. The β‐perylene is instead monomeric and involves conventional herringbone (HB) packing of the planar molecules. Basic structure types of polynuclear aromatic hydrocarbons were named in accordance with classification and rationalization proposed by Desiraju and Gavezzotti.[^37^, ^38^]

Oligorylenes have found many uses in pigments, dyes, organic photovoltaics and field‐effect transistors.^[39]^ In particular, terrylene is a very capable fluorophore, suitable for single molecule spectroscopy.[^40^, ^41^] Recently, there has been increasing interest in oligorylenes as singlet fission (SF) chromophores.[^42^, ^43^, ^44^, ^45^, ^46^, ^47^, ^48^] SF is the conversion of one photo‐generated singlet exciton into two triplet excitons,[^49^, ^50^, ^51^, ^52^, ^53^] which may significantly enhance the efficiency of solar cells by harvesting two charge carriers from one high‐energy photon, whose excess energy would otherwise be lost to heat.^[54]^ It has been suggested that terrylene may undergo SF, based on time‐dependent density functional theory (TDDFT) calculations of an isolated molecule.^[55]^

While both polymorphs of perylene and quaterrylene form well defined single crystals, terrylene typically forms small rectangular leaflets, which are too thin for typical single‐crystal XRD measurements. Also, no structure determination by powder XRD has been reported to date. Even if lattice constants and space group of terrylene were never measured experimentally, they have been tentatively extrapolated from those of SHB perylene and quaterrylene,^[33]^ and forecasted by crystal structure prediction.[^37^, ^38^] Here we report the successful application of 3D ED to terrylene, highlighting once more the potential of this method for total structure solution in cases where standard XRD methods are impractical.

The ability to solve the structure of crystals that are too small for standard optoelectronic characterisation calls for the implementation of new strategies, able to evaluate the solid‐state properties of nanomaterials. In this regard, *ab initio* simulations can deliver crucial information and allow the evaluation of a variety of fundamental properties, without any limit connected with crystal size. Utilising the so‐determined structure, we use many‐body perturbation theory calculations within the GW approximation and the Bethe‐Salpeter equation (BSE)[^56^, ^57^, ^58^, ^59^] to assess the potential of crystalline terrylene to undergo intermolecular SF in the solid‐state. Previously, crystalline quaterrylene has been identified as a promising SF candidate based on this method,^[60]^ while terrylene could not be assessed in the absence of a crystal structure.

## Methods

**Figure 2:**
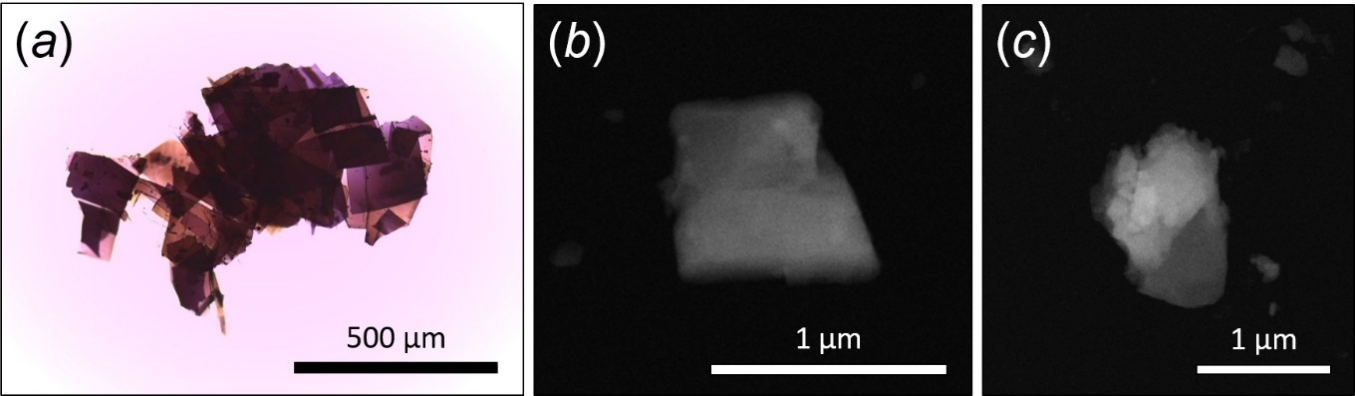
Terrylene sample under light microscopy (a) and HAADF‐STEM images (b, c) of two terrylene grains used for 3D ED data collection.

Crystal platelets (Figure 2a) were gently crushed and directly loaded on a carbon‐coated Cu TEM grid without any solvent or sonication. Terrylene was synthesised via two routes given by Buchta^[61]^ and Weiss.^[62]^ The keto carboxylic acid, from phthalic acid anhydride and naphthalene, was reduced with NaBH_4_ to 1‐naphthyl‐phtalide. This reacts with 1‐naphthyl‐Mg‐Br to give di‐1‐naphthyl‐isobenzofurane. Diels‐Alder reaction with acrylic acid, followed by elimination of H_2_O, gave cyano‐ or carboxy‐ternaphtyl. When submitted to an AlCl_3_/NaCl melt, the cyano adduct cyclized to a mixture of terrylene and cyano‐terrylene, the latter being easily removed by recrystallization owing to its higher solubility. By a similar treatment with AlCl_3_/NaCl, the carboxy‐ternaphtyl gave terrylene only. Small quantities of the nonalternant isomer (with one five‐membered ring) are effectively removed by fractional sublimation, chromatography, and recrystallization. The quaterrylene isomer is not formed in this synthesis. Both routes gave terrylene with the same spectral and structural characteristics.

High‐angle annular dark‐field scanning transmission electron microscopy (HAADF‐STEM) imaging and ED data were recorded with a Zeiss Libra 120 TEM operating at 120 kV and equipped with a LaB_6_ source. 3D ED was performed in STEM mode after defocusing the beam in order to have a pseudo‐parallel illumination on the sample. ED patterns were collected with a beam size of about 150–200 nm in diameter, obtained using a 5 μm C2 condenser aperture.^[63]^ Data were recorded using a single‐electron ASI MEDIPIX detector.^[64]^ Extremely low dose illumination was adopted in order to avoid beam damage.

3D ED acquisitions were performed, rotating the sample around the TEM goniometer axis in steps of 1°, in total tilt ranges up to 85°. Exposure time per frame was 1 s. Camera length was 180 mm, allowing resolution in real space up to 0.7 Å. After each tilt, a diffraction pattern was acquired, and the crystal position was tracked by STEM imaging. During the experiment, the beam was precessed around the optical axis by an angle of 1°. Precession was obtained using a Nanomegas Digistar P1000 device. All data acquisitions were performed at room temperature.

3D ED data were analysed using the software *PETS*.^[65]^ Structure determination was obtained by standard direct methods (SDM) as implemented in the software *SIR2014*.^[66]^ Data were treated with a fully kinematical approximation, assuming that *I_hkl_* was proportional to |*F_hkl_* |^2^. Kinematical least‐squares structure refinement was performed with the software *SHELXL* using electron atomic scattering factors.^[67]^ Dynamical structure refinement was performed with the software JANA2006.[^68^, ^69^, ^70^]

DFT structural optimisations of the terrylene crystal were carried out using two methods. Calculations using the Perdew‐Burke‐Ernzerhof based hybrid functional (PBE0)^[71]^ combined with the Grimme D3 pairwise dispersion method^[72]^ were conducted using the *CRYSTAL17* code,^[73]^ stopping when residual forces were below 1 meV/Å. A 4×4×4 k‐grid was used along with the all‐electron 6‐31(d) basis sets. Conformational relaxation of the asymmetric unit was allowed and confirmed that the structure sits at an energetic minimum. Calculations using the Perdew‐Burke‐Ernzerhof generalised gradient approximation (PBE)[^74^, ^75^] combined with the Tkatchenko‐Scheffler (TS) pairwise dispersion method^[76]^ were conducted using the *FHI‐aims* code.^[77]^ Tight numerical settings and tier 2 basis sets were used. Full unit cell relaxation was performed until no force component on any atom exceeded 0.01 eV/Å.

GW+BSE calculations were performed using the *BerkeleyGW* code.^[78]^ Quantum ESPRESSO^[79]^ was used to compute the mean‐field eigenvectors and eigenvalues and to generate the mean‐field coarse‐grid and fine‐grid wave functions with the PBE exchange‐correlation functional. We used a coarse k‐grid of 2×2×2 and a fine k‐grid of 4×4×4. Troullier‐Martins norm‐conserving pseudopotentials^[80]^ were used and the kinetic energy cut‐off was set to 50 Ry. About 550 unoccupied bands were included in the GW calculation. The static remainder correction was applied to accelerate the convergence with respect to the number of unoccupied states.^[81]^ The polarizability, inverse dielectric matrix, and GW self‐energy operator were constructed based on the mean‐field eigenvalues and eigenfunctions using the coarse k‐point settings. Optical properties, including excitation energies, exciton wave functions, and absorption spectra were calculated by solving the BSE within the Tamm‐Dancoff approximation (TDA). 24 valence bands and 24 conduction bands were included in the BSE calculation. Taking the full dielectric matrix as input to screen the attraction between the electron (e) and hole (h), the e–h interaction kernel was constructed on the coarse k‐point grid. To construct the Bethe–Salpeter Hamiltonian, the GW quasiparticle energies and e–h interaction kernel calculated with coarse k‐point settings were interpolated onto the fine k‐point grid. The subsequent diagonalization yielded the excitation energies and wave functions. The exciton wave functions were converged using a supercell of 4×10×4 based on the criterion proposed by Liu et al.^[82]^ The degree of singlet exciton charge transfer character (%CT) was calculated by double‐Bader analysis (DBA).[^60^, ^82^] The results for rubrene and pentacene are from Wang et al.,^[83]^ the results for quaterrylene, perylene, and tetracene are from Wang et al.,^[60]^ and the results for anthracene are from Liu et al.^[82]^

## 2 Results and Discussion

**Figure 3:**
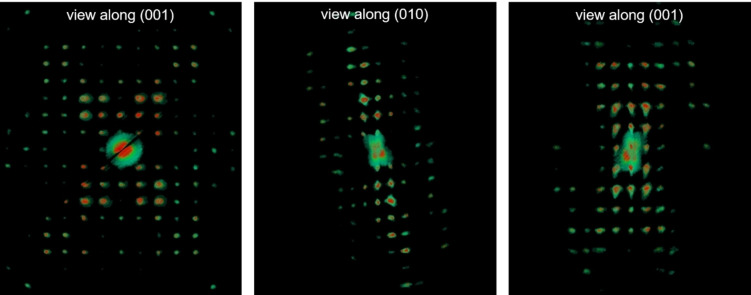
Reconstructions of 3D ED volume viewed along the main crystallographic directions.

3D ED data were recorded from seven crystal fragments with sizes less than 1 μm (Figure 2b, c), All datasets consistently showed the same primitive monoclinic unit cell, but only the two with the largest tilt ranges (70° and 80°, respectively) and without artefacts induced by polycrystallinity were selected for the accurate determination of cell parameters and for the integration of reflection intensities. Averaged cell parameters were *a*=11.4 Å, *b*=10.4 Å, *c*=14.4 Å and *β*=95.6°. The related cell volume would conveniently host four molecules of terrylene (*Z*=4). Extinction rules 0*k*0: *k*=2*n* and *h*0 *l*: *h*=2*n* were also observed (Figure 3), pointing unambiguously to space group *P*2_1_/*a* (14).

Reflection intensities extracted from both data sets were merged according to the observed Laue symmetry and a scale factor derived from the comparison of the strongest common reflections (which are assumed to be proportionally less affected by experimental errors and residual dynamical scattering).

Structure solution was performed by SDM and resulted in the automatic localization of all 30 non‐hydrogen atoms of the asymmetric unit. The structure of terrylene was kinematically least‐squares refined against 3D ED data, after imposing constraints on the aromatic rings and the assignment of all hydrogen atoms to calculated positions. The related Fourier map is shown in Figure S1. Eventually, a further refinement step was done taking into account dynamical scattering. More details about structure determination and refinement are reported in Supporting Information Table S1. Due to the small amount of sample and its extremely anisotropic crystal habit, the terrylene structure could not be validated by Rietveld refinement against powder XRD data, as has been proposed in previous works.[^20^, ^25^, ^26^] This is unfortunately a rather common situation for organic samples.[^22^, ^29^, ^84^]

**Figure 4:**
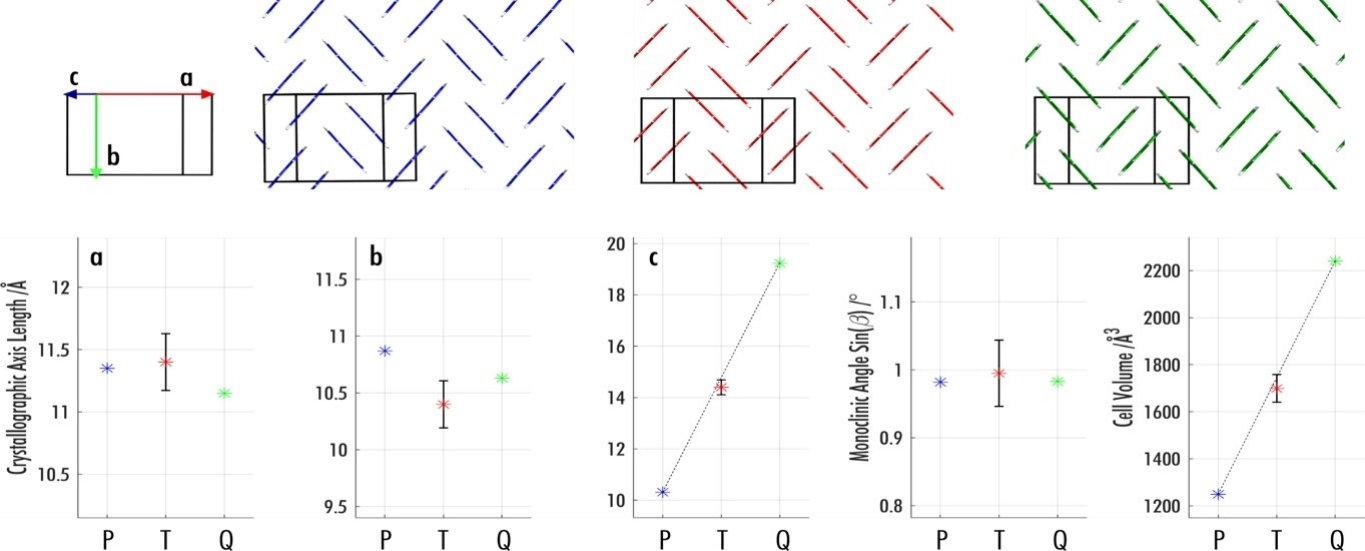
Packing (top) of SHB perylene (in blue), terrylene (in red) and quaterrylene (in green) and extrapolation of the crystallographic parameters (bottom) between all three structures.

Similarly, to SHB perylene and quaterrylene, the terrylene molecules arrange in dimers stacked in a SHB packing motif. The length of cell parameter *c* shows a clear trend through the three structures and relates to the increasing length of the backbones, which are almost perfectly oriented along this axis (Figure 4). Cell parameter *b* in terrylene is clearly shorter than for SHB perylene and quaterrylene. The cell volume is compensated however, by the monoclinic angle, which for terrylene is closer to 90°. No particular trend for the *a*‐axis could be observed. The SHB packing motif is characterized by strong intermolecular electronic coupling within a dimer, but weak coupling between dimers. This typically results in smaller band dispersion than in HB crystals, in which there is strong electronic coupling between π‐stacked molecules, as previously shown for the two polymorphs of perylene.^[60]^ The band structure of terrylene, shown in the SI, exhibits modest band dispersion, similar to quaterrylene and SHB perylene.^[60]^

**Figure 5:**
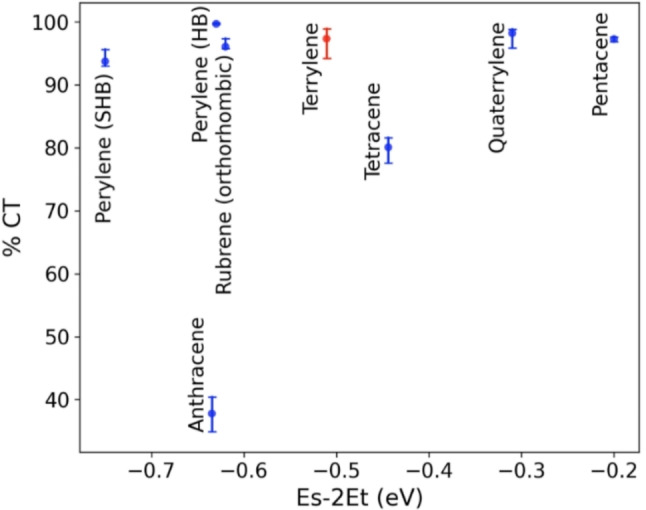
A two‐dimensional descriptor for assessing SF candidates. The thermodynamic driving force for SF (E_s_‐2E_t_) is displayed on the x‐axis and the singlet exciton charge transfer character (%CT) is displayed on the y‐axis. The error bars correspond to the range of %CT values obtained by using different hole positions when performing double‐Bader analysis. Terrylene, coloured in red, is compared to other oligorylene and acene crystals.

To assess the potential of terrylene to undergo SF in the solid‐state we evaluate it with respect to a two‐dimensional descriptor,[^60^, ^82^, ^85^, ^86^] as shown in Figure 5. The primary descriptor, displayed on the x‐axis, is the thermodynamic driving force for SF, which is the difference between the singlet exciton energy and twice the triplet exciton energy (E_s_‐2E_t_). A high driving force indicates that a material is likely to undergo SF with a high rate. However, an overly high driving force would lead to losses in solar energy conversion. Therefore, it has been suggested that materials with E_s_≈2E_t_ may be preferable.^[87]^ Owing to the approximations used in GW+BSE calculations, the values of E_s_‐2E_t_ are systematically underestimated. Hence, we restrict the discussion to qualitative comparisons between materials.

The secondary descriptor, displayed on the y‐axis, is the degree of charge transfer character (%CT) of the singlet exciton wave function. This descriptor is motivated by the growing body of experimental evidence for the involvement of an intermediate charge transfer state in the SF process.[^45^, ^51^, ^88^, ^89^, ^90^] A singlet exciton with a high degree of charge transfer character, i. e., with the hole and the electron located on different molecules, is thought to be favourable for SF.[^51^, ^91^, ^92^, ^93^, ^94^] The error bars correspond to the range of %CT values obtained for different hole positions within the double‐Bader analysis.[^60^, ^82^]

In Figure 5, the oligorylenes are compared to acenes with respect to these two descriptors. A similar trend of increasing SF driving force with backbone size is observed in both families. Pentacene has the highest SF driving force of the materials shown here and is known to undergo fast SF with a high triplet yield.[^95^, ^96^] Quaterrylene has been theoretically predicted to be close to pentacene,^[60]^ but this has yet to be experimentally verified. On the other end of the scale, anthracene, perylene, and their derivatives are known to undergo triplet‐triplet annihilation, the reverse process of SF, in which two triplet excitons are converted into one singlet exciton.^[97]^ Terrylene is positioned between rubrene and tetracene. SF has been experimentally observed in crystals of both rubrene[^98^, ^99^, ^100^, ^101^, ^102^, ^103^] and tetracene.[^104^, ^105^, ^106^, ^107^, ^108^, ^109^, ^110^] SF with a high triplet yield has also been observed in thin films of terrylene derivatives with different packing motifs.^[43]^ Based on this, terrylene is likely to undergo SF. GW+BSE yields an optical gap of 1.81 eV for crystalline terrylene, which is close to the optical gap of pentacene, as shown in Figure s S2 and S3. This means that terrylene could potentially absorb as much of the solar spectrum as pentacene and undergo SF with a smaller energy loss.

## 3 Conclusions

The crystal structure of terylene (cf. Cambridge Crystallographic Data Centre (CCDC) www.ccdc.cam.ac.uk/structures, deposition number 2086493), a well‐known capable fluorophore PAH suitable for spectroscopy, was successfully obtained by experimental diffraction data for the first time. Previously, structure solution by single‐crystal XRD was not possible due to limited crystal size and the thin leaflet morphology. Here, the structure was determined by direct methods on the basis of electron diffraction data collected from a single nanocrystal using the 3D ED approach. The stability of the result was confirmed by DFT structural optimizations. The successful structure determination of terrylene by 3D ED allowed for the evaluation of its excited‐state properties in the solid‐state using many‐body perturbation theory. Specifically, we were interested in assessing the potential of terrylene to undergo singlet fission in the solid state. Previously, in the absence of a crystal structure, promising calculations for an isolated molecule could not be extrapolated to the solid‐state. Here, we have compared terrylene to other oligorylene and acene crystals with respect to a two‐dimensional descriptor based on the thermodynamic driving force for SF and the degree of charge transfer character of the singlet exciton. Our results suggest that terrylene may be a potentially promising SF candidate.

## Conflict of interest

The authors declare no conflict of interest.

## Supporting information

As a service to our authors and readers, this journal provides supporting information supplied by the authors. Such materials are peer reviewed and may be re‐organized for online delivery, but are not copy‐edited or typeset. Technical support issues arising from supporting information (other than missing files) should be addressed to the authors.

Supporting Information

Supporting Information
