# Supplementary material for: 3D Electron Diffraction Structure Determination of Terrylene, a Promising Candidate for Intermolecular Singlet Fission
Source: Chemphyschem. 2021 Jun 30;22(15):1631–7. doi: 10.1002/cphc.202100320 (PMC8457070; doi:10.1002/cphc.202100320)
Supplement: Supplementary file 1 — Supporting Information [file CPHC-22-1631-s001.pdf]

ChemPhysChem

Supporting Information

3D Electron Diffraction Structure Determination of Terrylene, a Promising Candidate for Intermolecular Singlet Fission

Charlie L. Hall, Iryna Andrusenko, Jason Potticary, Siyu Gao, Xingyu Liu, Werner Schmidt, Noa Marom, Enrico Mugnaioli, Mauro Gemmi,* and Simon R. Hall*

Supplementary Information

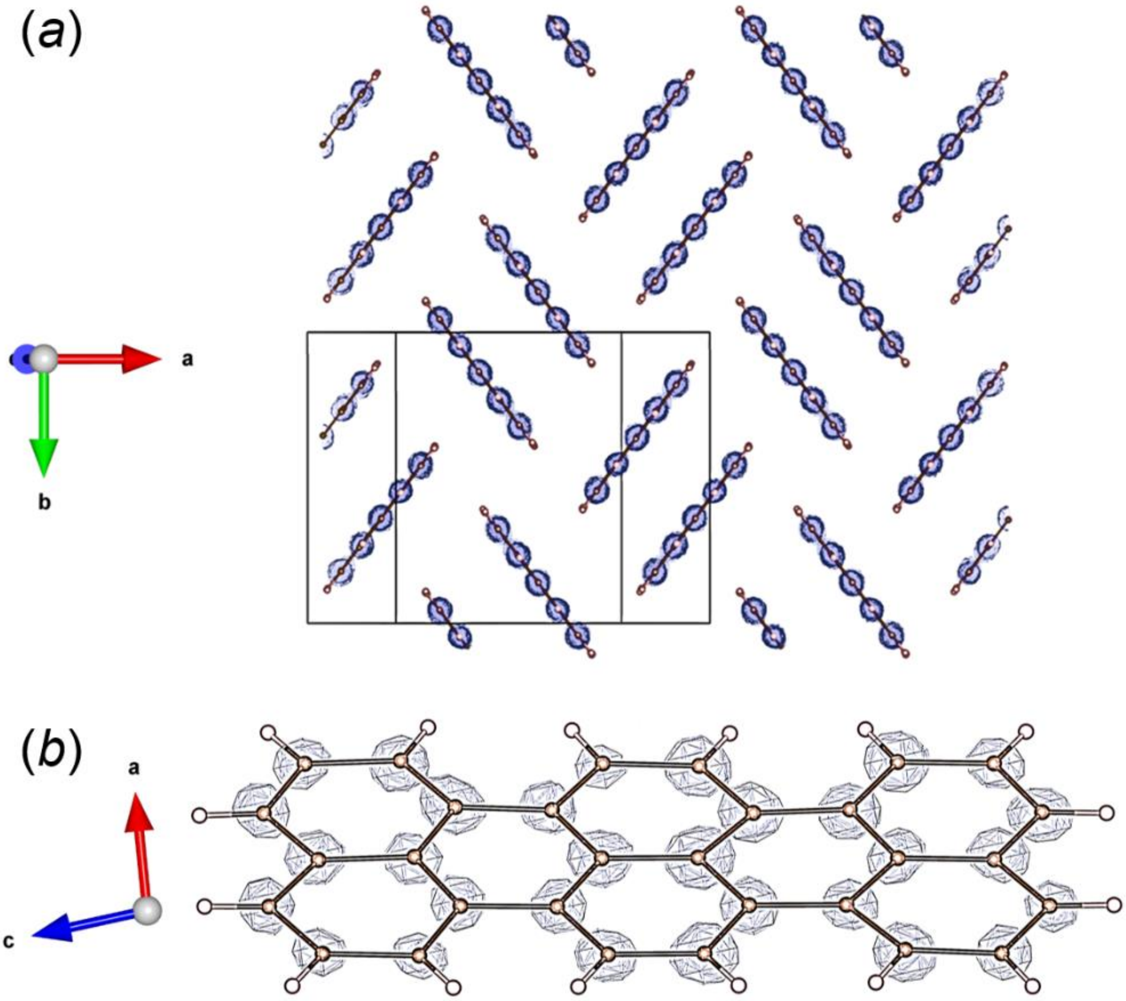

Figure S1: Terrylene structure and Fourier map: (a) view down the sandwich-herringbone packing and (b) one separated terrylene molecule. Deposition Number 2086493 contains the supplementary crystallographic data for this paper. These data are provided free of charge by the joint Cambridge Crystallographic Data Centre and Fachinformationszentrum Karlsruhe Access Structures service www.ccdc.cam.ac.uk/structures.

Table S1: Selected parameters from structure solution (*SIR2014*), and kinematical (*SHELXL*) and dynamical (*JANA2006*) refinements based on the 3D ED data

Crystallographic information	
Asymmetric unit content	$C_{30}H_{16}$
Z	4
Space group	$P2_1/a$
a (Å)	11.4(2)
b (Å)	10.4(2)
c (Å)	14.4(3)
β (°)	95.6(5)
Volume (Å^3)	1699(59)
Structure solution (*SIR2014*)	
Data resolution (Å)	0.9
No. of sampled reflections	5836
No. of independent reflections	2311
Independent reflections coverage (%)	95
Global thermal factor U_{iso} (Å^2)	0.01919
R_{int} (*F*) (%)	20.30
R_{SIR} (%)	34.85
Kinematical refinement (*SHELXL*)	
Data resolution (Å)	0.9
R_{int} (F^2) (%)	23.50
No. of reflections (all)	2311
No. of reflections (> 4σ)	1433
*R*1 (all) (%)	44.68
*R*1 (4σ) (%)	41.07
Goodness-of-fit	2.671
Dynamical refinement (*JANA2006*)	
g(max) (Å^{-1})	1.5
Maximal S_g^0 (Matrix) (Å^{-1})	0.01
Maximal S_g^0 (refine) (Å^{-1})	0.1
RS_g	0.4
No. of integration steps	96
No. of zones	72
Calculated thickness (Å)	446
R (obs) (%)	13.29
w*R* (all) (%)	16.39
Goodness-of-fit	2.35

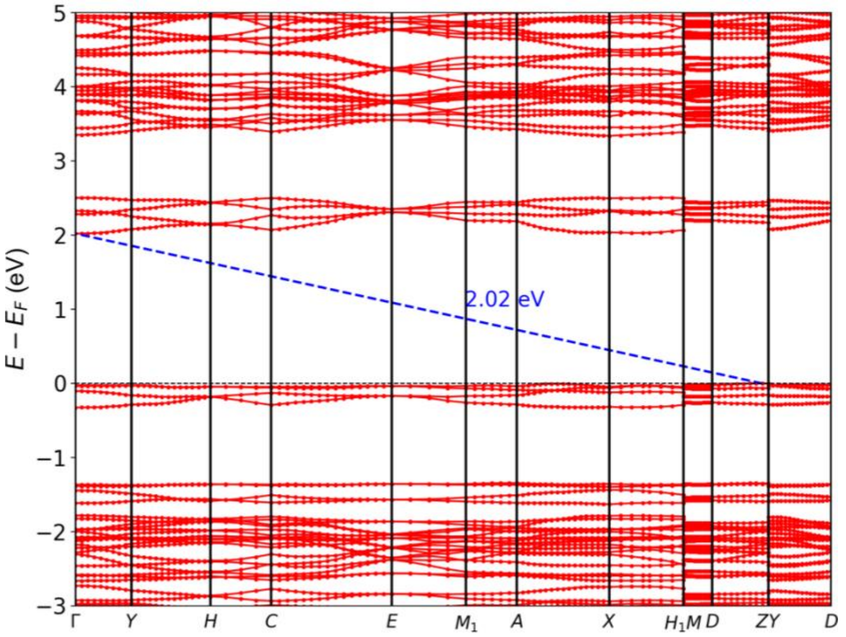

Figure S2: Quasi-particle band structure of terrylene calculated by GW@PBE. The valence band maximum is shifted to zero. The fundamental gap is also shown.

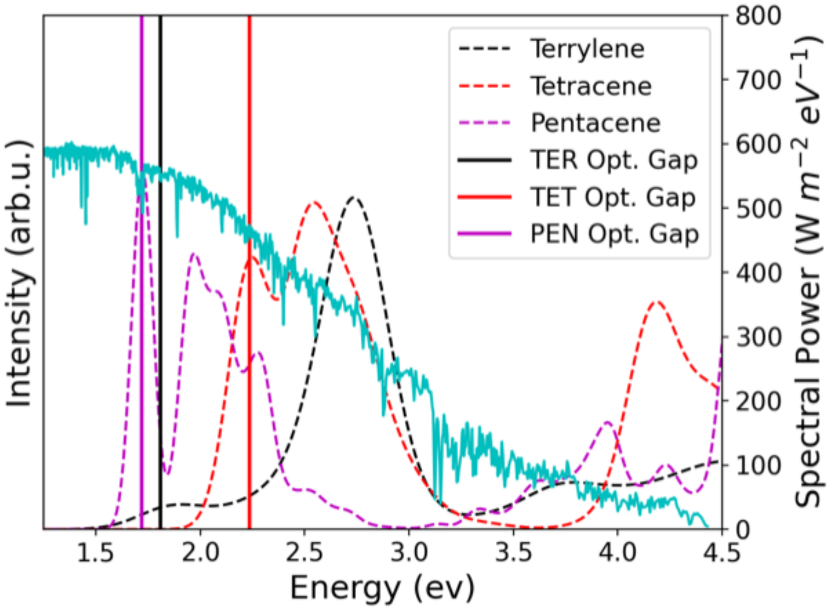

Figure S3: Comparison between GW+BSE@PBE absorption spectra of crystalline terrylene, tetracene, and pentacene. The blue line is the solar spectrum[1]. The optical gaps are indicated by the vertical lines.

1. Gueymard, C. A. Parameterized transmittance model for direct beam and circumsolar spectral irradiance. *Sol. Energy* **71**, (2001).
